# Evaluation of upper airways after bimaxillary orthognathic surgery in patients with skeletal Class III pattern using cone-beam computed tomography

**DOI:** 10.1590/2177-6709.21.1.034-041.oar

**Published:** 2016

**Authors:** Marília Spínola Azevêdo, Andre Wilson Machado, Inêssa da Silva Barbosa, Lucas Senhorinho Esteves, Vanessa Álvares Castro Rocha, Marcos Alan Vieira Bittencourt

**Affiliations:** 1 Specialist in Orthodontics, Universidade Federal da Bahia (UFBA), Salvador, Bahia, Brazil; 2 Adjunct professor, Universidade Federal da Bahia (UFBA), Department of Orthodontics, Salvador, Bahia, Brazil; 3 Master in OMFS, Unigranrio, Rio de Janeiro, Rio de Janeiro, Brazil and Fellowship in OMFS, Oklahoma University, Norman, Oklahoma, USA; 4 Residency in OMFS, Universidade de São Paulo (USP), Bauru, SP, Brazil and Northwestern University, Evanston, Illinois, USA; 5 Associate professor, Universidade Federal da Bahia (UFBA), Department of Orthodontics, Salvador, Bahia, Brazil. Director, Brazilian Board of Orthodontics and Dentofacial Orthopedics (BBO)

**Keywords:** Airway remodeling, Orthognathic surgery, Obstructive sleep apnea, Cone-beam computed tomography

## Abstract

**Introduction:**

It has been suggested that mandibular setback surgery, combined or not with maxillary advancement as a treatment alternative for patients with mandibular prognathism, can induce changes in upper airway space (UAS). Therefore, this study aimed to assess the response of the upper airway in the oropharynx region of patients with Class III skeletal pattern that underwent bimaxillary orthognathic surgery (maxillary advancement and mandibular setback) combined with mentoplasty.

**Material and Methods:**

The sample comprised 26 cone-beam computed tomography (CBCT) scans of 13 patients. The examination was taken before and after surgery. UAS volume, sagittal area, length and minimal axial area with its width, depth and location, were measured with the aid of Dolphin Imaging^TM^software version 11.5 Premium. Data were statistically treated by applying Shapiro-Wilk test and Student's paired t-test, considering as statistically significant the results of which p-value was lower than 0.05.

**Results:**

No statistically significant differences were found in any measurements evaluated.

**Conclusions:**

No significant changes were observed in the oropharynx after bimaxillary orthognathic surgery and mentoplasty.

## Abstract (pt)

**Introdução:**

tem sido sugerido que a cirurgia de recuo mandibular - que é uma das alternativas de tratamento para pacientes com prognatismo mandibular -, associada ou não ao avanço maxilar, pode promover alterações no espaço das vias aéreas superiores. Por esse motivo, o presente estudo teve como objetivo avaliar o comportamento das vias aéreas superiores, na região da orofaringe, em pacientes com padrão esquelético de classe III submetidos à cirurgia ortognática bimaxilar (avanço maxilar e recuo mandibular) e mentoplastia.

**Métodos:**

a amostra foi composta por 26 tomografias computadorizadas de feixe cônico de 13 pacientes, tomadas antes e após o procedimento cirúrgico. Usando o programa Dolphin Imaging^(r)^ versão 11.5 Premium, foram mensurados o volume do espaço orofaringeano; sua área (em um corte sagital); a mínima secção transversal dessa região, com sua largura, profundidade e localização; além de seu comprimento. Os dados obtidos foram tratados estatisticamente, tendo-se utilizado os testes Shapiro-Wilk e T-student pareado, considerando-se significativos os resultados com p < 0,05.

**Resultados:**

não foram encontradas diferenças estatisticamente significativas em qualquer uma das medidas avaliadas.

**Conclusão:**

não foram observadas modificações significativas na orofaringe após a realização da cirurgia ortognática bimaxilar e mentoplastia.

## INTRODUCTION

Class III skeletal pattern is characterized by disharmony in the anteroposterior direction, with maxillary deficiency, mandibular excess or both. In cases in which orthodontic compensation is not possible, orthodontic-surgical treatment is a therapeutic alternative, usually mandibular setback combined or not with maxillary advancement.^1^

After setback, mandibular spatial position is shifted to a region closer to the posterior pharyngeal wall. This new relation to the composing structures of the upper airways may compromise air space and predispose the individual to developing obstructive sleep apnea-hypopnea (OSAH), also leading to alteration of other structures, such as hyoid bone and palatal soft tissue, thus narrowing posterior air space (PAS) to a greater extent.^2^

OSAH is a disorder characterized by recurrent episodes of partial or complete obstruction of upper airway during sleep.^3^Patients with this condition show a higher risk of developing cardiovascular diseases, including hypertension, angina, heart attack and stroke.^4^^,^ 5 Mandibular advancement surgery, combined or not with maxillary advancement aiming at increasing upper airways space, is among a wide range of treatment alternatives proposed in the literature.^6^^,^ 7 On the other hand, some studies have shown narrowing of PAS after mandibular setback surgery, but on a lesser extend in cases in which setback is associated with maxillary advancement. Advancement of the soft palate and velopharyngeal muscles caused by Le Fort I osteotomy and anterior maxillary replacement may explain the reduced constriction effect caused by mandibular setback surgery.^8^^,^ 9 ^,^ 10

According to Lee et al,^11^ bimaxillary orthognathic surgery for maxillary advancement and mandibular setback does not affect total airway volume. The results disclosed by the authors increased volume in the upper region and decreased volume in the lower region. Thus, there was compensation of the total volume, which, according to the authors, would not have occurred if only posterior repositioning of the mandible had been performed.

Another aspect to be taken into consideration is the combined rotational movement of the occlusal plane. Studies show that when counterclockwise rotation movement is performed during maxillo-mandibular advancement, an increase in PAS occurs.^12^^,^ 13 However, when the same occlusal plane rotation is performed with maxillary advancement and mandibular setback, there is narrowing of airspace.^12^

There has been a growing interest in upper airway space evaluation due to reports of post-surgery symptoms that appear when isolated mandibular setback is performed, such as snoring and narrowing of PAS, which are associated with OSAH.^8^ However, most upper airway evaluations derive from measurements taken on bidimensional radiographic exams.^14^^,^ 15 ^,^ 16 Therefore, in order to achieve higher reliability, it is mandatory to conduct studies using tridimensional analysis carried out by means of cone-beam computed tomography.

## MATERIAL AND METHODS

This research was approved by Universidade Federal da Bahia, School of Dentistry, Institutional Review Board. It should be noted that all patients signed a free and informed consent form authorizing the use of their examinations, performed exclusively for therapeutic purposes, in this research.

This is a retrospective, longitudinal and quantitative study. CBCT scans of 13 patients were used. All of them had skeletal Class III pattern, according to Wits analysis, with values ranging from -2.4 mm to -17.5 mm. The sample was composed by eight male and five female subjects that had passed through the pubertal spurt phase with ages between 17 and 40 years old. All patients had undergone bimaxillary orthognathic surgery (maxillary advancement and mandibular setback) and mentoplasty with the use of rigid internal fixation. All patients had CBCT scans taken before and after surgery. The same team of surgeons, headed by a chief surgeon, performed all surgeries. It is important to highlight that although all patients underwent maxillary advancement, nine of them required lower repositioning, while three individuals required upper repositioning of the maxilla. Counterclockwise rotation of the maxillomandibular complex was performed in 10 patients. All patients had mentoplasty performed, whereas upper anterior repositioning was performed in seven patients, isolated upper repositioning in three and advancement alone in other three individuals by means of the basilar sliding osteotomy technique. Patients who had a history of craniofacial syndromes and those whose examinations made it difficult to visualize the anatomical structures were excluded from the study.

All CT scans were obtained with the aid of i-CAT^TM^ system (Imaging Sciences International, Hatfield, PA, USA), with acquisition protocol set at 120 Kvp; 36.9 mA; 0.4 mm voxel; FOV 22 cm and rotation time of 40 seconds. CT scans were performed with the patient seated, Frankfort horizontal plane parallel to the ground, and in maximum and habitual intercuspation. All patients were instructed to keep their tongues at rest during examination.

Digital Imaging and Communications in Medicine (DICOM) files were imported and a tridimensional reconstruction of maxillary structures was performed by Dolphin Imaging^TM^ software version 11.5 Premium (Dolphin Imaging & Management Solutions, Chatsworth, California, USA). CT scans were obtained before surgery (T_0_) and between the range of four to six months after surgery (T_1_).

Before measurements, the digital position of the head was standardized according to the axial, coronal and sagittal planes. In lateral view, right Orbital and Porion points were located and positioned, so as to coincide with Frankfort horizontal plane. In frontal view, the points of the right and left frontozygomatic sutures were marked and the median line of the software placed exactly on the median line of the patient.

**Figure 1:**
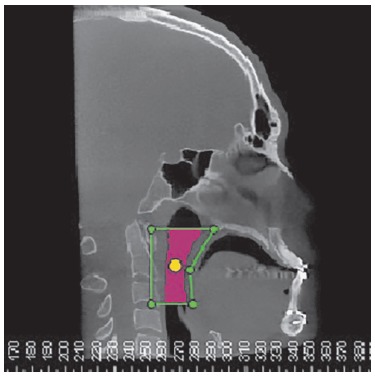
- Defining upper airspace in the sagittal plane and positioning the main marker.

**Figure 2:**
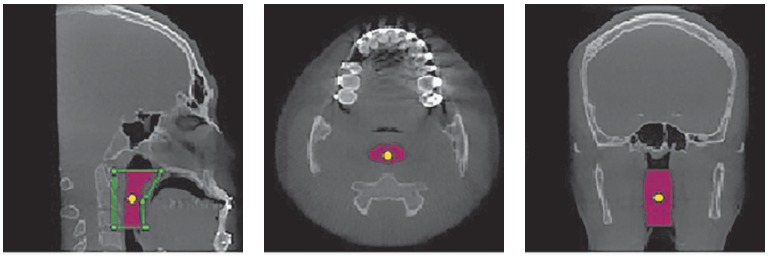
- Area determined by the main marker in three multiplanar sections (sagittal, axial and coronal).

After digital head positioning, the software sinus/airway tool, by means of which the software provides the user with the image of a median sagittal section, was used. This image was used to determine the space of the oropharynx, while lines passing respectively by the Posterior Nasal Spine point and the apex of the epiglottis parallel to the Frankfort horizontal plane demarcated the upper and lower limits. The anterior and posterior limits were established so that a prism was formed with the upper and lower lines, thereby encompassing the entire oropharynx. The main marker was placed within the delimited space by filling the whole area (Fig 1). The other multiplanar images (axial and coronal) were checked, so as to verify that the main marker encompassed the whole area (Fig 2).

Thereafter, using specific Dolphin tools, the following measurements were taken:

1. Sagittal area (SA) recorded in mm^2^.
2. Airway volume (Vol) recorded in mm^3^.
3. Minimum axial area (MAA) recorded in mm^2^. For this measurement, the lower limit was displaced 5 mm above the apex of the epiglottis.
4. MAA depth (MAAD) recorded in mm. It is the distance between the posterior and anterior walls of the oropharynx in the MAA region performed on the sagittal midline.
5. MAA width (MAAW) also recorded in mm. It is the distance between the lateral walls of the oropharynx in the MAA region on the coronal plane.
6. MAA location (Loc) recorded in mm. To determine this location, the distance between the Sella point and the MAA was measured on the sagittal section.
7. Airway length (C) recorded in mm. It is the distance between the upper and lower limits of the oropharynx on the sagittal section.

**Figure 3:**
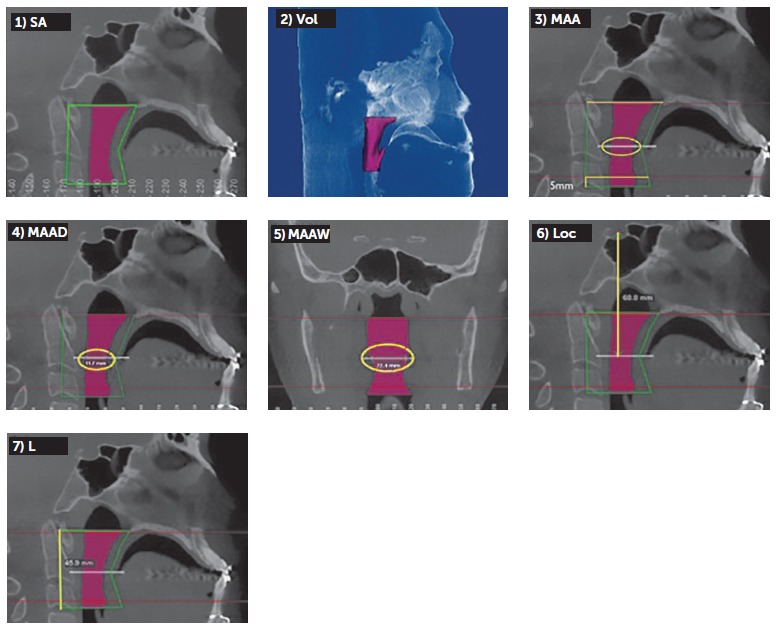
- Representations of all measures: 1) sagittal area (SA), 2) airway volume (Vol), 3) minimum axial area (MAA), 4) MAA depth (MAAD), 5) MAA width (MAAW), 6) MAA location (Loc), 7) airway length (L).

Sagittal and coronal sections used to implement these measures were automatically provided by the software in the greatest constriction area. Representations of all measures can be seen in Figure 3.

Prior to measuring, five CT scans were randomly selected in order to calibrate the examiner. All measurements were carried out in two stages with an interval of two weeks in between them, under the same conditions. For all variables, random error was calculated according to Dahlberg's formula (S²=Σd²/2n) in order to verify intraexaminer agreement. Analysis of measurement reproducibility was performed by testing intraclass correlation, both with a confidence level of 95%. A high reliability rate was found, since all coefficients were greater than or equal to 0.84 for both time points. Thereafter, measurements were taken on all CT scans obtained before surgery (T_0_) and after surgery (T_1_). Data were statistically treated. Shapiro-Wilk test was applied and results showed that the values ​​had non-normal distribution. Thereafter, Student's paired t-test was used. All results with p-value lower than 0.05 were considered statistically significant.

## RESULTS

As previously reported, sample was composed by CT scans of patients aged between 17 and 40 years old, with a mean age of 26 years and standard deviation of seven years, as shown in Table 1.

**Table 1:** - Mean, standard deviation, maximal value and minimal value of patients' ages.

	**Age**
Mean	26
Standard deviation	7
Maximal value (Vmax)	40
Minimal value (Vmin)	17

It is noteworthy that, in comparing the preoperative cephalometric tracings with the postoperative tracings, it was found that patients who took part in this study experienced a mean maxillary advancement of 3.35 mm and a mean mandibular setback of 3.92 mm, which produced a mean sagittal change of 7.27 mm in the maxillo-mandibular relationship (Table 2).

**Table 2:** - Mean, standard deviation, maximal value and minimal value of maxillary advancement and mandibular setback performed on patients.

	**Max. advancement**	**Mand. setback**
Mean	3.35	3.92
Standard deviation	1.97	2.39
Maximal value (Vmax)	8	6
Minimal value (Vmin)	1	0.5

Table 3 shows the mean and standard deviation of each variable measured before (T_0_) and after (T_1_) surgery. As it can be seen, there was no statistically significant difference in any of them (*p* > 0.05). 

**Table 3:** - Mean, standard deviation (SD) and p-value for each measurement, pre (T_0_) and post (T_1_) operative.

	**T_0_**	**T_1_**	**p-value**		
	**Mean**	**SD**	**Mean**	**SD**	
Vol (mm^3)^	15856.762	6945.6809	16657.138	5588.4639	0.487
SA (mm^2)^	637.315	181.997	674.1	148.02174	0.217
MAA (mm^2)^	200.423	84.9928	210.192	88.8093	0.642
MAAD (mm)	9.692	2.5825	11.615	5.3797	0.194
MAAW (mm)	22.446	6.7441	22.046	5.025	0.703
Loc (mm)	74.015	12.0402	72.238	15.0019	0.737
L (mm)	52.131	8.9496	51.985	6.9349	0.933

## DISCUSSION

Anteroposterior skeletal disharmony in Class III skeletal pattern patients may be surgically treated with maxillary advancement, mandibular setback or a combination of both. The type of surgery to be performed will depend on the location and the amount of discrepancy, also considering facial aesthetics.^1^ Many times, maxillary advancement is chosen based upon potential impairment of upper airways. However, in cases of larger skeletal discrepancies or those in which there is greater aesthetic impairment by mandibular influence, it becomes unavoidable to perform mandibular setback. In the present study, mean total discrepancy was 7.27 mm, which justified the need for bimaxillary surgery. It is important to highlight that one patient required turbinectomy and septoplasty, since there was a 5-mm upper repositioning of the maxilla, and interference of nasal turbines would hinder final bone position, which could lead to septum deviation and functional alteration.

For proper airway evaluation, linear, surface and volumetric measurements are required. In the present study, all these measurements were taken on CT scans which provide more accurate anatomical information and are more suitable for evaluation of upper airway tests than conventional radiography.^17^

The literature on the effects of orthognathic surgery on airway space is controversial. In this study, it was observed that surgery induced a slight increase in upper airway volume, although no statistically significant difference was found (*p* = 0.487), which corroborates the study by Jakobsone et al^18^ who used CT scans of ten patients and also reported a slight increase, albeit not statistically significant. On the other hand, Park et al^20^ found no difference in the total volume of airways, although they did find a decrease in the oropharyngeal region. In other study evaluating women, no significant changes in volume of the pharyngeal airway were found.

By separately evaluating the upper and lower portions of the airways, it was found that maxillary advancement enlarged the upper region whereas mandibular setback reduced the lower region, in what appears to be a compensatory process.^11^ This fact may justify why there was no significant alteration in any of the measurements taken in this study, in the sense that there may have been some compensation in the values found, since all patients had undergone maxillary advancement and mandibular setback surgery.

On the other hand, in a study evaluating a sample of nine individuals who had undergone bimaxillary surgery, a decrease in the volume of the pharynx was noted.^10^ Their sample, however, differed from the present study sample, since patients did not undergo advancement mentoplasty. It is known that this procedure can by itself cause expansion of upper airways and has been proposed in the literature as a treatment for mild to moderate OSAH.^21^ Given that, in the present sample, all patients underwent mentoplasty, thus, it is believed that this might have influenced the results. Contradicting this information, in a study comparing patients who had undergone mentoplasty with patients who had not, no statistically significant difference was found between them in terms of pharyngeal airway volume.^22^

Polysomnography and cephalometric radiographs of pre- and postoperative patients without OSAH who had undergone mandibular setback were evaluated, and it was concluded that some narrowing of the upper airway had occurred, but with no evidence that these patients had acquired sleep disorders. According to the authors, the airways of individuals with a skeletal Class III pattern display wider dimensions than individuals whose values are normal; after surgery, however, these values remain within normality range.^23^

Another study showed the influence of the type of movement performed during bimaxillary surgery, rotational or not, which affects airway behavior. According to the authors, if counterclockwise rotation is performed, there will be an increase in the size of airspace. This should therefore be, whenever possible, the movement of choice.^13^ In this study, this aspect was not taken into account, as the number of patients who had undergone bimaxillary surgery without rotation was too small, which made it difficult to compare the groups.

Another aspect evaluated in this investigation was the area of upper airway in a sagittal section. It was observed that there was a slight increase, although not statistically significant (*p* = 0.217), from 637.3 mm^2^ to 674.1 mm^2^. This finding is consistent with another research which evaluated cephalometric radiographs of 48 patients divided into three groups, one of which comprised patients who had undergone bimaxillary surgery. The authors found no significant differences in the oropharyngeal and hypopharyngeal regions, which together comprised the same area used in this work, i.e., from the Posterior Nasal Spine to the apex of the epiglottis.^24^

This study measured the area, depth, width and location of the major airway narrowing region, also known as minimum axial area (MAA). This assessment is paramount, since airway collapse occurs due to resistance to airflow.^25^ It was reported that average cross-section is much smaller in apneic patients compared to healthy patients.^26^Galvin et al^27^ found an average of 134.2 mm^2^in healthy individuals. In this study, after comparing preoperative *versus* postoperative CT scans, a mean of 200.4 mm^2^ was found for the former and 210.2 mm^2^ for the latter. These values are well above the range described for patients with OSAH, i.e., approximately 50 mm^2^ or less.^28^ It is noteworthy that none of the patients assessed in this study showed any values ​​lower than this. Also worthy of note is the fact that, in order to identify the MAA, the lower limit of the airway was displaced, so that it reached 5 mm above the apex of the epiglottis, once we observed, during preliminary studies, that this measurement was always demarcated by the software close to the epiglottis region, making it clear that the end of this structure hindered the formation of a more constricted region.

As it can be seen in Table 3, the minimal axial area had its size and depth slightly increased, while its width decreased, and the MAA was displaced upwards. However, these measures were not statistically significant. Additionally, it can be observed that a slight increase in depth was responsible for a slight increase in ​​MAA, revealing that even after mandibular setback, the region of greatest constriction showed a slight gain in the anteroposterior direction. This was probably due to the advancement of the maxilla and chin and counterclockwise rotation of the maxilla. Furthermore, slight upward displacement was not expected, since it was assumed that mandibular setback would decrease the upper airway space, which in turn would shift the area of greatest constriction down to a lower region.

In a similar study, no statistically significant difference was found in the area, depth and width of the cross-sections studied.^10^ In another study, two groups were evaluated; one had undergone mandibular setback while the other one underwent bimaxillary surgery. The authors observed a decrease in the cross-sectional area of both groups, but found no statistically significant difference in the latter.^9^

According to Segal et al,^29^ patients diagnosed with OSAH exhibit a correlation between airway length and OSAH severity to such an extent that, due to air resistance, the greater the length of the airway, the greater the respiratory disturbance index. For this reason, it is important to evaluate this measurement. In the present study, this measurement remained virtually unchanged, showing a slight, not statistically significant (*p* = 0.933) decrease. Other study, however, found a significant decrease in the length of the pharynx after bimaxillary surgery.^10^

Many studies show that mandibular setback can change the dimensions of upper airways and predispose one to OSAH.^2^^,^ 8 ^,^ 30 Other studies show that, in comparing mandibular setback surgery with bimaxillary orthognathic surgery, a greater decrease occurs in the airways if only mandibular setback surgery is performed.^9^^,^ 10 ^,^ 19 Thus, in view of the results found in this investigation, one can conclude that when treating patients with skeletal Class III pattern, bimaxillary orthognathic surgery (maxillary advancement and mandibular setback) does not lead to significative dimensional alterations in the oropharynx region.

## CONCLUSIONS

In view of the results achieved in this study, it can be concluded that there are no significant changes in the upper airway of patients with skeletal Class III pattern after bimaxillary orthognathic surgery and mentoplasty.

## References

[1] Boeck EM, Lunardi N, Pinto AS, Pizzol KEDC, Boeck RJN (2011). Occurrence of skeletal malocclusions in Brazilian patients with dentofacial deformities. Braz Dent J.

[2] Demetriades N, Chang DJ, Laskarides C, Papageorge M (2010). Effects of mandibular retropositioning, with or without maxillary advancement, on the oro-naso-pharyngeal airway and development of sleep-related breathing disorders. J Oral Maxillofac Surg.

[3] Masood A, Phillips B (2000). Sleep apnea. Curr Opin Pulm Med.

[4] Harding SM (2000). Complications and consequences of obstructive sleep apnea. Curr Opin Pulm Med.

[5] Phillips BG, Somers VK (2002). Sleep disordered breathing and risk factors for cardiovascular disease. Curr Opin Pulm Med.

[6] Riley RW, Powell NB, Guilleminault C (1989). Inferior mandibular osteotomy and hyoid myotomy suspension for obstructive sleep apnea: a review of 55 patients. J Oral Maxillofac Surg.

[7] Sibel AEL, Hakan EL, Palomo JM, Baur DA (2011). A 3-dimensional airway analysis of an obstructive sleep apnea surgical correction with cone beam computed tomography. J Oral Maxillofac Surg.

[8] Chen F, Terada K, Hua Y, Saito I (2007). Effects of bimaxillary surgery and mandibular setback surgery on pharyngeal airway measurements in patients with Class III skeletal deformities. Am J Orthod Dentofacial Orthop.

[9] Degerliyurt K, Ueki K, Hashiba Y, Marukawa K, Nakagawa K, Yamamoto E (2008). A comparative CT evaluation of pharyngeal airway changes in Class III patients receiving bimaxillary surgery or mandibular setback surgery. Oral Surg Oral Med Oral Pathol Oral Radiol Endod.

[10] Hong JS, Park YH, Kim YJ, Hong SM, Oh KM (2011). Three-dimensional changes in pharyngeal airway in skeletal class III patients undergoing orthognathic surgery. J Oral Maxillofac Surg.

[11] Lee Y, Chun YS, Kang N, Kim M (2012). Volumetric changes in the upper airway after bimaxillary surgery for skeletal Class III malocclusions: a case series study using 3-dimensional cone-beam computed tomography. J Oral Maxillofac Surg.

[12] Mehra P, Downie M, Pita MC, Wolford LM (2001). Pharyngeal airway space changes after counterclockwise rotation of the maxillomandibular complex. Am J Orthod Dentofacial Orthop.

[13] Zinser MJ, Zachow S, Sailer HF (2013). Bimaxillary 'rotation advancement' procedures in patients with obstructive sleep apnea: a 3-dimensional airway analysis of morphological changes. Int J Oral Maxillofac Surg.

[14] Kawakami M, Yamamoto K, Fujimoto M, Ohgi K, Inoue M, Kirita T (2005). Changes in tongue and hyoid positions, and posterior airway space following mandibular setback surgery. J Craniomaxillofac Surg.

[15] Pereira-Filho VA, Castro-Silva LM, de Moraes M, Gabrielli MF, Campos JA, Juergens P Cephalometric evaluation of pharyngeal airway space changes in Class III patients undergoing orthognathic surgery (2011). J Oral Maxillofac. Surg.

[16] Ribeiro CO, Bittencourt MAV, Brandão RA (2011). Avaliação dos efeitos da cirurgia ortognática de recuo mandibular isolado e combinado no tamanho da orofaringe. Ortho Sci Orthod Sci Pract.

[17] Lenza MG, Lenza MM, Dalstra M, Melsen B, Cattaneo PM (2010). An analysis of different approaches to the assessment of upper airway morphology: a CBCT study. Orthod Craniofac Res.

[18] Jakobsone G, Neimane L, Krumina G (2010). Two- and three-dimensional evaluation of the upper airway after bimaxillary correction of Class III malocclusion. Oral Surg Oral Med Oral Pathol Oral Radiol Endod.

[19] Park SB, Kim YI, Son WS, Hwang DS, Cho BH (2012). Cone-beam computed tomography evaluation of short- and long-term airway change and stability after orthognathic surgery in patients with Class III skeletal deformities: bimaxillary surgery and mandibular setback surgery. Int J Oral Maxillofac Surg.

[20] Panou E, Motro M, Ates M, Acar A, Erverdi N (2013). Dimensional changes of maxillary sinuses and pharyngeal airway in Class III patients undergoing bimaxillary orthognathic surgery. Angle Orthod.

[21] Foltán R, Hoffmannová J, Pretl M, Donev F, Vlk M (2007). Genioglossus advancement and hyoid myotomy in treating obstructive sleep apnoea syndrome: a follow-up study. J Craniomaxillofac Surg.

[22] Kim MA, Kim BR, Youn JK, Kim YJ, Park YH (2014). Head posture and pharyngeal airway volume changes after bimaxillary surgery for mandibular prognathism. J Craniomaxillofac Surg.

[23] Hochban W, Schürmann R, Brandenburg U, Conradt R (1996). Mandibular setback for surgical correction of mandibular hyperplasia - does it provoke sleep-related breathing disorders. Int J Oral Maxillofac Surg.

[24] Aydemir H, Memikoglu U, Karasu H (2012). Pharyngeal airway space, hyoid bone position and head posture after orthognathic surgery in Class III patients. Angle Orthod.

[25] Abramson Z, Susarla SM, Lawler M, Bouchard C, Troulis M, Kaban LB (2011). Three-dimensional computed tomographic airway analysis of patients with obstructive sleep apnea treated by maxillomandibular advancement. J Oral Maxillofac Surg.

[26] Lan Z, Itoi A, Takashima M, Oda M, Tomoda K (2006). Difference of pharyngeal morphology and mechanical property between OSAHS patients and normal subjects. Auris Nasus Larynx.

[27] Galvin JR, Rooholamini SA, Stanford W (1989). Obstructive sleep apnea: diagnosis with ultrafast CT. Radiology.

[28] Avrahami E, Englender M (1995). Relation between CT axial cross-sectional area of the oropharynx and obstructive sleep apnea syndrome in adults. AJNR Am J Neuroradiol.

[29] Segal Y, Malhotra A, Pillar G (2008). Upper airway length may be associated with the severity of obstructive sleep apnea syndrome. Sleep Breath.

[30] Kawamata A, Fujishita M, Ariji Y, Ariji E (2000). Three-dimensional computed tomographic evaluation of morphologic airway changes after mandibular setback osteotomy for prognathism. Oral Surg Oral Med Oral Pathol Oral Radiol Endod.

